# Draft Genome Sequences of Two Chemosynthetic Arcobacter Strains Isolated from Hydraulically Fractured Wells in Marcellus and Utica Shales

**DOI:** 10.1128/genomeA.00159-18

**Published:** 2018-05-17

**Authors:** Jenny Panescu, Rebecca A. Daly, Kelly C. Wrighton, Paula J. Mouser

**Affiliations:** a Department of Civil, Environmental, and Geodetic Engineering, The Ohio State University, Columbus, Ohio, USA; b Department of Microbiology, The Ohio State University, Columbus, Ohio, USA

## Abstract

Genome sequences were obtained for two isolates of the genus Arcobacter from saline fluids produced from hydraulically fractured shale gas wells in the Marcellus and Utica formations. These genomes provide insight into microbial sulfur cycles occurring in a high-salt deep terrestrial shale environment.

## GENOME ANNOUNCEMENT

Natural gas and crude oil represent the two leading forms of hydrocarbon resources produced in the United States (1). Hydraulic fracturing is a technique used to increase hydrocarbon yield from formations with low permeability, such as black shales, involving the high-pressure injection of fluids into the deep subsurface to establish new fractures for hydrocarbon release through a wellbore (2). In this engineered system, microbial processes are implicated in infrastructure damage and well souring (3); thus, understanding the dominant microbial metabolisms is important for improving wellbore longevity and maximizing hydrocarbon yield. The bacterial strains Arcobacter marinus UTICA-S4D1 and MARC-MIP3H16 were isolated from highly saline (88 to 120 ppm total dissolved solids) produced fluids from two distinct hydraulically fractured shale gas wells in the Utica-Point Pleasant (Ohio) and Marcellus (West Virginia) formations, respectively. Arcobacter, a member of the class *Epsilonproteobacteria*, is a Gram-negative non-spore-forming curved rod found in a wide range of food, clinical, and marine environments (4–7), including other hydrocarbon-producing wells (8, 9). Here, we describe the genomic sequencing of two Arcobacter strains and their relevance as satellite taxa to the fractured shale ecosystem.

These Arcobacter isolates were cultivated from produced fluids collected at 122 days (Utica) and 70 days (Marcellus) after flowback began under a low-oxygen environment on Difco Marine agar 2216 at 5% NaCl held at 30°C. Genomic DNA was isolated using a Qiagen DNeasy kit (Hilden, Germany) and sequenced at the Joint Genome Institute, Walnut Creek, CA, USA. Genome assemblies were constructed from Illumina MiSeq data (SPAdes version 3.6.2) and annotated using the Integrated Microbial Genomes platform (Pipeline version 4.12.1). The assemblies generated 69 and 70 contigs for UTICA-S4D1 and MARC-MIP3H16, respectively. UTICA-S4D1 and MARC-MIP3H16 have 2,779 and 2,845 protein-coding genes, with G+C contents of 27.12% and 27.18%, respectively. A ribosomal protein S3 comparison indicated that these two Arcobacter strains are highly similar, with 98.6% average nucleotide identity (ANI).

These Arcobacter strains are facultative aerobes capable of utilizing amino acids, propionate, and tricarboxylic acid (TCA) cycle intermediates, but not carbohydrates, for carbon and energy. Additionally, each genome held the potential for autotrophic carbon fixation (10), as well as the ability to reduce nitrate (*napAB*) and nitroalkanes (*nmo*) to ammonia (*nrfAH*) for energy generation. Both Arcobacter strains contained genes for sulfur oxidation from thiosulfate (*soxABCD* and *soxYZ*), tetrathionate (*ttr*), and sulfide (*sqr*), which produce corrosive agents commonly detected in shale-produced fluids (3). These strains adapt to high salinities using a K^+^ and Na^+^ salt-in strategy or through the acquisition and/or production of the organic osmolytes ectoine and glycine betaine. The isolation of Arcobacter marinus UTICA-S4D1 and Arcobacter marinus MARC-MIP3H16 during the first 3 months of shale gas production indicates the short-lived presence of oxygen or nitrate for chemosynthesis and the oxidation of reduced sulfur species in these saline fractured systems.

### Accession number(s).

These whole-genome sequences for MARC-MIP3H16 and UTICA-S4D1 have been deposited in ENA under the accession numbers PTIW01000000 and FUYO01000000 and can be assessed at JGI Integrated Microbial Genomes and Microbiome Samples under the IMG genome identification (ID) numbers 2703719342 and 2700989666, respectively.
